# How effective is the BNT162b2 mRNA vaccine against SARS-CoV-2 transmission and infection? A national programme analysis in Monaco, July 2021 to September 2022

**DOI:** 10.1186/s12916-024-03444-6

**Published:** 2024-06-05

**Authors:** Thomas Althaus, Christopher E. Overton, Isabelle Devaux, Thomas House, Arnaud Lapouze, Alexa Troel, Bertrand Vanzo, Margaux Laroche, Alexandre Bordero, Pernille Jorgensen, Richard Pebody, Eric J. Voiglio

**Affiliations:** 1 Directorate of Health Affairs, Monaco, Monaco; 2 Department of Mathematical Sciences, University of Liverpool, Liverpool, UK; 3 United Kingdom Health Security Agency, London, UK; 4 World Health Organization Regional Office for Europe, Copenhagen, Denmark; 5 Department of Mathematics, University of Manchester, Manchester, UK; 6 Digital Service Department, Monaco, Monaco

**Keywords:** SARS-CoV-2; COVID-19; Vaccine effectiveness; Transmission

## Abstract

**Background:**

We quantified SARS-CoV-2 dynamics in different community settings and the direct and indirect effect of the BNT162b2 mRNA vaccine in Monaco for different variants of concern (VOC).

**Methods:**

Between July 2021 and September 2022, we prospectively investigated 20,443 contacts from 6320 index cases using data from the Monaco COVID-19 Public Health Programme. We calculated secondary attack rates (SARs) in households (*n* = 13,877), schools (*n* = 2508) and occupational (*n* = 6499) settings. We used binomial regression with a complementary log–log link function to measure adjusted hazard ratios (aHR) and vaccine effectiveness (aVE) for index cases to infect contacts and contacts to be infected in households.

**Results:**

In households, the SAR was 55% (95% CI 54–57) and 50% (48–51) among unvaccinated and vaccinated contacts, respectively. The SAR was 32% (28–36) and 12% (10–13) in workplaces, and 7% (6–9) and 6% (3–10) in schools, among unvaccinated and vaccinated contacts respectively. In household, the aHR was lower in contacts than in index cases (aHR 0.68 [0.55–0.83] and 0.93 [0.74–1.1] for delta; aHR 0.73 [0.66–0.81] and 0.89 [0.80–0.99] for omicron BA.1&2, respectively). Vaccination had no significant effect on either direct or indirect aVE for omicron BA.4&5. The direct aVE in contacts was 32% (17, 45) and 27% (19, 34), and for index cases the indirect aVE was 7% (− 17, 26) and 11% (1, 20) for delta and omicron BA.1&2, respectively. The greatest aVE was in contacts with a previous SARS-CoV-2 infection and a single vaccine dose during the omicron BA.1&2 period (45% [27, 59]), while the lowest were found in contacts with either three vaccine doses (aVE − 24% [− 63, 6]) or one single dose and a previous SARS-CoV-2 infection (aVE − 36% [− 198, 38]) during the omicron BA.4&5 period.

**Conclusions:**

Protection conferred by the BNT162b2 mRNA vaccine against transmission and infection was low for delta and omicron BA.1&2, regardless of the number of vaccine doses and previous SARS-CoV-2 infection. There was no significant vaccine effect for omicron BA.4&5. Health authorities carrying out vaccination campaigns should bear in mind that the current generation of COVID-19 vaccines may not represent an effective tool in protecting individuals from either transmitting or acquiring SARS-CoV-2 infection.

**Supplementary Information:**

The online version contains supplementary material available at 10.1186/s12916-024-03444-6.

## Background

Although SARS-CoV-2 has increased its intrinsic infectiousness since it first emerged in 2019 [1, 2], there is strong evidence that most vaccines continue to be effective in reducing severe disease and mortality, including for the latest omicron subvariants [3]. Whether these vaccines can still stop viral transmission and infection, however, remains unclear and as noted by the World Health Organization (WHO): “more evidence is needed to determine exactly how well they stop infection and transmission” [4]. When COVID-19 vaccines first became available, national campaigns tended to present these vaccines as an effective tool to “stop the virus” (Additional Fig. S1) [5, 6]. Calculating the impact of vaccination on transmission is key to inform optimal vaccination policies, but relies on a number of conditions: (i) both index cases and contacts should be included regardless of symptoms; (ii) factors influencing viral transmission should be accounted for, such as the circulating SARS-CoV-2 variant(s), number of vaccine doses, prior SARS-CoV-2 infection, age, and context in which transmission takes place [7, 8]. Only a limited number of studies that have adjusted for such factors and collected data from real-life settings, testing all contacts and collecting their results, regardless of symptoms exist [9–15]. Within the Monaco COVID-19 Public Health Programme (MCPHP), all contacts from a RT-PCR SARS-CoV-2 confirmed index case were identified by contact tracing and prospectively followed up. Based on this national programme, we quantified viral transmission for SARS-CoV-2 delta (B.1.617.2) and omicron subvariants (BA.1, BA.2, BA.4 and BA.5) in different settings, according to index case and contact vaccination and infection status between July 2021 and September 2022. We also measured contacts’ risk of infection and index cases’ infectiousness, using an adjusted statistical analysis.

## Methods

### Study setting

With a total of 36,297 inhabitants living on a 2.02 square kilometre territory, Monaco is the most densely populated country in the world, with a median age of 46.4 years [16]. Demographic and health system characteristics are similar to neighbouring larger western European countries. Every day, workers commute to Monaco, mostly from France and Italy, doubling the local population [17].

The Monaco COVID-19 Public Health Programme (MCPHP) offers free-of-charge SARS-CoV-2 screening and vaccination services to all residents and workers, in a unique community health centre. No consent form nor justification to benefit from these services was required, as this is considered a national public health programme. Screening consists of nasopharyngeal (NP) and salivary reverse-transcriptase polymerase chain reaction (RT-PCR)-testing. Additional SARS-CoV-2 screening includes rapid antigen tests (RATs) validated by the French National Authority for Health, available in Monaco pharmacies [18]. COVID-19 vaccination was launched on the 31st of December 2020 based on the WHO recommendations. The programme exclusively relied on the Pfizer mRNA vaccine [19]. The MCPHP also included an epidemiological component with prospective contact tracing. The epidemiological team contained three distinct units, according to the context of the contact: occupational medicine; school medicine; and the epidemiological monitoring unit (households; private meetings; travelling; health settings; restaurants and others).

### Study design and population

Individuals attending the COVID-19 community centre were asked to provide their full name, date of birth, phone number, email address and presence of symptoms by the community centre staff, which was directly recorded in the national MCPHP dataset. Molecular SARS-CoV-2 analysis was carried out by the national laboratory (at the Monaco Scientific Centre), and results were automatically uploaded in the national MCPHP dataset. For those attending Monaco pharmacies for rapid antigen testing (Ag-RDT), results were manually entered in the national MCPHP dataset by the community centre’s staff, with the same information regardless of nationality. Every day, the epidemiological team of the MCPHP gathered all SARS-CoV-2 positive cases from the national dataset, contacting them by phone and email. These index cases were asked to provide information on all contacts from 2 days prior to the positive test or symptom onset (if any), including the “risk type” (high risk if contact of at least 15 min within 2 m from a confirmed case without protective measures; or low risk if contact with protective measures), date, and the context in which contact tool place (school; household, etc.) [20]. Contacts were required to provide the same information as index cases by phone and email and to be tested. In case the SARS-CoV-2 test and/or vaccine injections (if any) were not performed in Monaco and thus not in the national MCPHP dataset, contacts were asked to provide a proof of test and/or vaccination that was manually recorded in the dataset.

Contacts were requested to be tested following the Monaco national policy on COVID-19 [21]:

- In household, test on the same day as the index case, and again at day 5 if negative;
- Outside household, test at day 5 following the last contact with the index case;
- The contacts’ test results (PCR test or RAT) were automatically recorded in the MCPHP dataset if carried out in Monaco; and
- Contacts tested outside Monaco were asked to provide evidence of the test. In case of missing test, the epidemiological team was in charge of following up with the contact.

The MCPHP dataset was extracted between July 2021 and September 2022 for this study.

### Definitions

Index case:

- First date of onset in the study setting;
- A positive documented test (RT-PCR test or RAT) in Monaco;
- Residents, or non-residents working or studying in Monaco regardless of age; and
- Excluded if previously tested positive within the past 60 days [22].

Contact:

- As identified by the index case, with either a documented RT-PCR test or RAT result within 14 days following the last contact with the index case;
- In household, contacts who tested positive on the same day as index cases with the same date of symptom onset (if any) were classified co-primary cases and not contacts;
- Contacts with date of symptom onset ≥ one day after the index case were considered secondary cases; and
- Non-household contacts who tested positive on the same day as index cases were considered co-primary cases and not contacts, regardless of the date of symptom onset (if any).

Vaccination status:

- Individuals who received a vaccine injection within 14 days before their positive SARS-CoV-2 test were considered to hold their previous vaccination status, except for booster doses that were considered to be effective seven days after injection (e.g. a contact who received a third dose of vaccine 3 days prior to their test was considered to have received only two-vaccine doses at the time of the test) [23];
- “Fully vaccinated” was defined as:
  - A two-vaccine dose regimen; or
  - A single vaccine dose and a previously documented SARS-CoV-2 infection; and
  - At least one booster dose.
- “Unvaccinated” individuals defined as:
  - No vaccine injection with or without medical contra-indication.

### Data processing

We renamed and recoded the variables to ensure all variable names and values were in an appropriate format for analysis. One case may have several contacts and the records related to each case were repeated by the number of contacts for each case. We created a unique record ID, where the first 4 digits were for the case ID, followed by the letter K and the last two digits were for the contact ID.

**Fig. 1:**
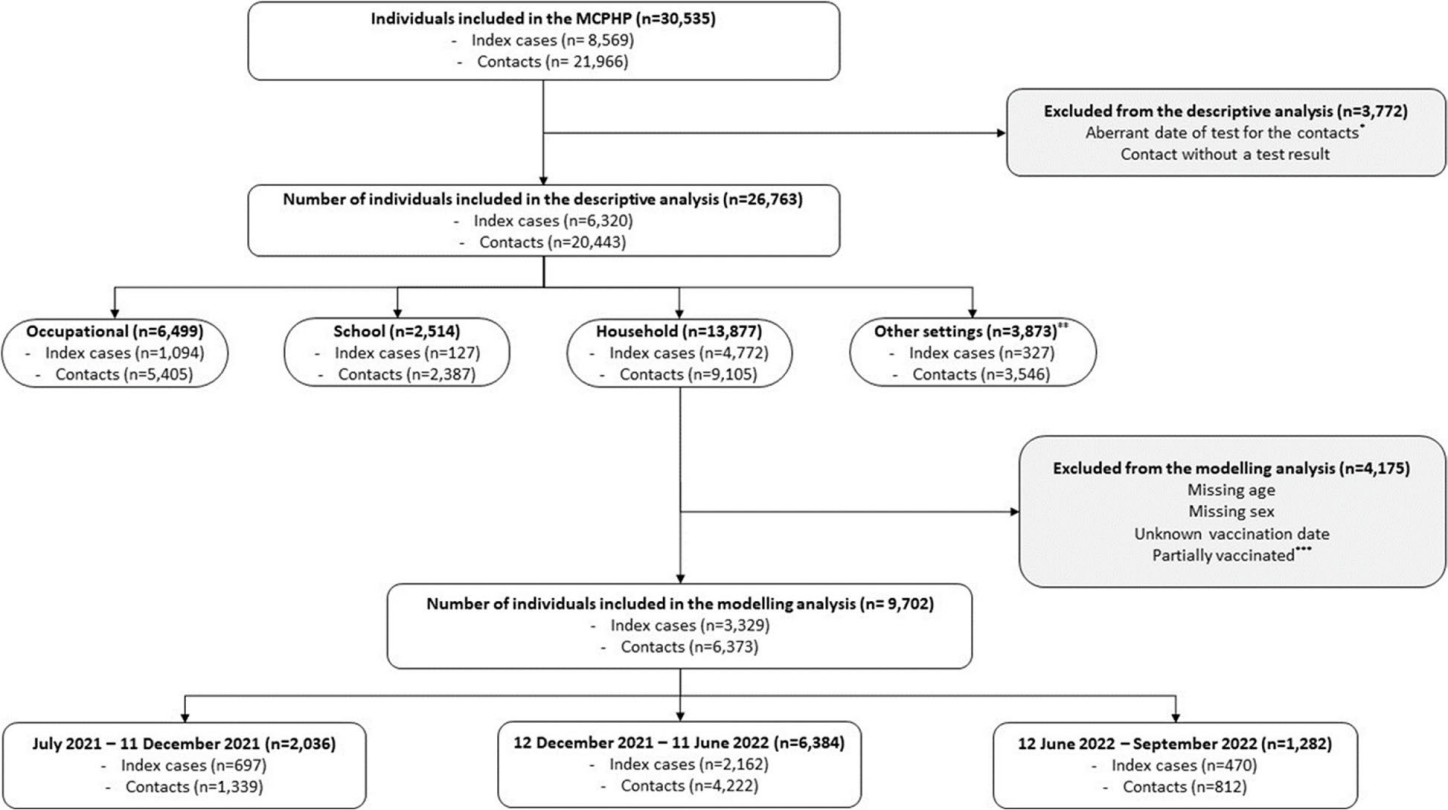
Flowchart for descriptive and modelling analysis. *Difference in the date of the test between index cases and contacts should be between 1 and 14. In household, the difference can be 0–14 days, and contacts were considered co-primary cases if tested positive on the same day than the index case and if similar date of symptom onset. **Other settings of contamination included private meetings; travelling; restaurants; healthcare settings; and unknown places of contamination. ***Co-circulation Delta-Omicron period corresponded to December 2021. ****Partially vaccinated corresponded to a single dose of vaccine at least 14 days prior to the test without a previous documented SARS-CoV-2 infection

The initial dataset was checked for completeness and data quality issues as described in Fig. 1.

We described characteristics of index cases and contacts by setting, including household, school and occupational (Additional table S1).

We measured the Secondary Attack Rate (or SAR) as the proportion of contacts infected in each setting 1–14 days after exposure to a primary case. SAR among contacts was calculated in vaccinated and unvaccinated cases and for different time periods corresponding to VOC predominance. For the calculation of vaccine effectiveness (VE), we performed the following:

1. Removed all records where the date of vaccination was not available (*n* = 649);
2. Removed records with unknown test results (*n* = 1771);
3. Ensured all dates were correctly formatted and fixed any clearly incorrect dates (e.g. dates in the future);
4. Assigned age groups: 0–16, 17–39, 40–59, 60 + , and removed records where age was unknown (*n* = 47);
5. Removed data with unknown gender (*n* = 64);
6. Corrected inconsistent vaccination dates (e.g. the third vaccine injection is dated before the second one);
7. Assigned vaccination status to individuals based on vaccinations received at least 14 days prior to the contact event;
8. Defined the following vaccination groups: unvaccinated — no vaccines; vaccination one unique dose — one vaccination dose with a SARS-CoV-2 documented prior infection; two doses — two vaccination doses; three doses — three or more vaccination doses;
9. Removed records with other vaccination histories (*n* = 202); and
10. Defined variant time periods.

The majority of excluded records were missing information on vaccination history or test results. Since these were the two main variables of interest in our study, we did not perform imputation, to avoid making assumptions about the nature of the missing records. The majority of the missing tests were “not done”, suggesting they are likely missing at random. Therefore, removing these data points should not bias the analysis.

VE was first calculated considering all fully vaccinated individuals regardless of the vaccination scheme (two-vaccine dose regimen, single vaccine dose and a previously documented SARS-CoV-2 infection, at least one booster dose) as one group (aggregate vaccination histories). The objective was to provide an overview of the vaccine’s effectiveness regardless of the number of doses received and potential previous SARS-CoV-2 infection. Then, we refined the analysis for each vaccination scheme (disaggregate vaccination histories). Analysing disaggregate vaccination schemes aimed to evaluate the impact of the vaccine alone with two doses, with and without a booster dose, and with a previous SARS-CoV-2 infection [24, 25].

### Statistical analysis

We carried out this analysis in the MCPHP for all index cases with a documented positive test (RT-PCR test or RAT) in Monaco, and for all contacts with a documented test (RT-PCR test or RAT), from June 2021 to September 2022. Over this time, the SARS-CoV-2 variant predominance changed, from delta, followed by omicron BA.1 and BA.2, followed by omicron BA.4 and BA.5. To define appropriate time periods where the different variants were dominant, we carried out linear discriminant analysis using the R package MASS [26] on a subset of data for which genomically confirmed lineages were available; this achieved an accuracy of over 93%, thus we used time as an instrumental variable to identify predominant lineage. This analysis identified three time periods: 23/06/2021 to 11/12/2021 corresponded to delta variant predominance; 12/12/2021 to 11/06/2022 corresponded to omicron BA.1 and BA.2 predominance; and 12/06/2022 to 28/09/2022 corresponded to omicron BA.4 and BA.5 predominance (Additional Fig. S2).

Descriptive statistics for continuous variables with normal distribution used means and standard deviations (SD) and medians with inter-quartile ranges (IQR) for non-normally distributed continuous variables. Comparisons between groups used *t*-tests for normally distributed variables, the Mann–Whitney test for non-normally distributed variables, and th echi-squared test for categorical variables.

We measured the secondary attack rate (SAR) overall, defined as the probability that a *possible* secondary case (i.e. contact of index case) is actually detected as a secondary case, for the location of exposure (i.e. in household, workplace and school contexts), and according to vaccination and infection status. We applied multiple binomial regression to the contact events data. A value of 0 indicated no infection within 1–14 days, and a value of 1 indicated infection within 1–14 days. In our regression, we adjusted for age, gender, and vaccination status of index cases and contacts, and stratified the effect of vaccination by the dominant SARS-CoV-2 variant in circulation at the time of the contact event by modelling an interaction between the circulating variant and the vaccination status. We modelled age using four broad age categories: 0–16, 17–39, 40–59, 60 + years. We used a binomial error structure to capture the binary nature of the outcome variable. However, instead of the most commonly used logit link function used in binomial regression, we used a complementary log–log (*cloglog*) link function. This link function was chosen since it allowed us to transform the modelled effect sizes into hazard ratios rather than odds ratios, which can be more directly linked both to secondary attack rates and vaccine effectiveness. To calculate the adjusted hazard ratio (aHR), we calculated the exponent of the modelled effect size. From this, the adjusted vaccine effectiveness (aVE) was calculated as one minus the aHR. Analysis was performed using R version 4.2.0 in Rstudio version 2023.03.0 [27, 28].

## Results

### Secondary attack rate

Of 30,535 individuals registered in the epidemiological component of the MCPHP between July 2021 and September 2022, 26,763 were kept in the descriptive analysis, including 6320 index cases and 20,443 contacts (Fig. 1). Amongst 5852 index cases with available vaccination data, 2614 (44.7%)) were not vaccinated and 3121 (52.3%)) were fully vaccinated, including 1534 (26.2%) with two doses, 1465 (25.0%) with at least one booster dose, and 122 (2.1%) with a single dose and a previous documented SARS-CoV-2 infection. Amongst 16,618 contacts with available vaccination data, 7449 (44.8%) were not vaccinated and 8910 (53.6%) were fully vaccinated, including 4323 (26.0%) with two doses, 4145 (24.9%) with at least one booster dose, and 442 (2.7%) with a single dose and a previous documented SARS-CoV-2 infection.

Households were the setting with the highest SAR: regardless of the vaccination status of index cases, 55% (95% confidence interval 54, 57) and 50% (48–51) of unvaccinated and vaccinated contacts were infected, respectively, between June 2021 and September 2022 (Table 1). Among unvaccinated contacts, the SAR was 57% (55–49) and 51% (48–54) in unvaccinated and vaccinated index cases, respectively, while in vaccinated contacts, the SAR was 48% (45–51) and 51% (49–53) in unvaccinated and vaccinated index cases, respectively. The difference in SAR between unvaccinated and vaccinated contacts decreased alongside variant emergence (50% *versus* 37% in delta; 60% *versus* 53% in omicron BA.1&2; 41% *versus* 49% in omicron BA.4&5). The highest SAR was calculated at 62% (59–64), in both unvaccinated index cases and contacts during the omicron BA.1&2 period.

**Table 1:** Secondary attack rate (SAR, 95% CI) by setting and VOC period in Monaco between July 2021 and September 2022

	**Household**							**Workplace**							**School**						
	**Index cases**	**Contacts**	**Infected**	**Total**	**SAR**	**95% CI**		**Index cases**	**Contacts**	**Infected**	**Total**	**SAR**	**95% CI**		**Index cases**	**Contacts**	**Infected**	**Total**	**SAR**	**95% CI**	
**July 2021 to September 2022** ** (all variants)**	**All** ** (*n* = 4427)**	NV	1714	3093	55%	54%	57%	**All** ** (*n* = 798)**	NV	194	604	32%	28%	36%	**All** ** (*n* = 113)**	NV	103	1374	7%	6%	9%
		FV	1723	3472	50%	48%	51%		FV	193	1666	12%	10%	13%		FV	12	190	6%	3%	10%
	**Not vaccinated** ** (*n* = 2036)**	NV	1202	2119	57%	55%	59%	**Not vaccinated** ** (*n* = 298)**	NV	58	181	32%	25%	39%	**Not vaccinated** ** (*n* = 98)**	NV	70	1095	6%	5%	8%
		FV	562	1176	48%	45%	51%		FV	38	329	12%	8%	15%		FV	8	135	6%	2%	10%
	**Fully vaccinated** ** (*n* = 2391)**	NV	432	846	51%	48%	54%	**Fully vaccinated** ** (*n* = 500)**	NV	36	224	16%	11%	21%	**Fully vaccinated** ** (*n* = 15)**	NV	7	169	4%	1%	7%
		FV	1096	2154	51%	49%	53%		FV	82	1028	8%	6%	10%		FV	0	46	0%	0%	0%
**July 2021 to 11 December 2021** ** (delta)**	**All** ** (*n* = 958)**	NV	404	806	50%	47%	54%	**All** ** (*n* = 322)**	NV	71	182	39%	32%	46%	**All** ** (*n* = 95)**	NV	67	1096	6%	5%	8%
		FV	186	500	37%	33%	41%		FV	39	337	12%	8%	15%		FV	7	169	4%	1%	7%
	**Not vaccinated** ** (*n* = 670)**	NV	332	657	51%	47%	54%	**Not vaccinated** ** (*n* = 197)**	NV	52	116	45%	36%	54%	**Not vaccinated** ** (*n* = 81)**	NV	61	946	6%	5%	8%
		FV	110	275	40%	34%	46%		FV	25	172	15%	9%	20%		FV	7	126	6%	2%	10%
	**Fully vaccinated** ** (*n* = 288)**	NV	72	149	48%	40%	56%	**Fully vaccinated** ** (*n* = 125)**	NV	19	66	29%	18%	40%	**Fully vaccinated** ** (*n* = 14)**	NV	6	150	4%	1%	7%
		FV	76	225	34%	28%	40%		FV	14	165	8%	4%	13%		FV	0	43	0%	0%	0%
**12 December 2021 to 11 June 2022** ** (omicron BA.1&2)**	**All** ** (*n* = 2765)**	NV	1096	1837	60%	57%	62%	**All** ** (*n* = 308)**	NV	11	160	7%	3%	11%	**All** ** (*n* = 17)**	NV	10	168	6%	2%	10%
		FV	1223	2322	53%	51%	55%		FV	54	683	8%	6%	10%		FV	1	12	8%	− 7%	24%
	**Not vaccinated** ** (*n* = 1165)**	NV	771	1252	62%	59%	64%	**Not vaccinated** ** (*n *= 58)**	NV	3	51	6%	− 1%	12%	**Not vaccinated** ** (*n* = 16)**	NV	9	149	6%	2%	10%
		FV	408	789	52%	48%	55%		FV	11	115	10%	4%	15%		FV	1	9	11%	− 9%	32%
	**Fully vaccinated** ** (*n* = 1600)**	NV	325	585	56%	52%	60%	**Fully vaccinated** ** (*n *= 250)**	NV	8	109	7%	2%	12%	**Fully vaccinated** ** (*n* = 1)**	NV	1	19	5%	− 5%	15%
		FV	815	1533	53%	51%	56%		FV	43	568	8%	5%	10%		FV	0	3	0%	0%	0%
**12 June 2022 to September 2022** ** (omicron BA.4&5)**	**All** ** (*n* = 697)**	NV	131	317	41%	36%	47%	**All** ** (*n* = 165)**	NV	11	61	18%	8%	28%							
		FV	245	504	49%	44%	53%		FV	26	336	8%	5%	11%							
	**Not vaccinated** ** (*n* = 198)**	NV	96	206	47%	40%	53%	**Not vaccinated** ** (*n* = 42)**	NV	2	13	15%	− 4%	35%							
		FV	43	111	39%	30%	48%		FV	2	42	5%	− 2%	11%							
	**Fully vaccinated** ** (*n *= 499)**	NV	35	111	32%	23%	40%	**Fully vaccinated** ** (*n* = 123)**	NV	9	48	19%	8%	30%							
		FV	202	393	51%	46%	56%		FV	24	294	8%	5%	11%							

*FV* fully vaccinated, including previously documented SARS-CoV-2 infection and one vaccine dose; two vaccine doses; at least one booster dose, *NV* non-vaccinated

At the workplace, the SAR was higher in unvaccinated than vaccinated contacts between July 2021 and September 2022: 32% (28–36) *versus* 12% (10–13), respectively. Similarly to households, this difference reduced alongside variant emergence (39% *versus* 12% in delta; 7% *versus* 8% in omicron BA.1&2; and 18% *versus* 8% in omicron BA.4&5). The difference in SAR between vaccinated and unvaccinated index cases was also reduced alongside variant emergence.

In schools, the overall SAR was the lowest of all studied settings, without marked differences between unvaccinated and vaccinated contacts between July 2021 and September 2022: 7% (6–9) *versus* 6% (3–10), respectively.

### Household vaccine effectiveness

**Fig. 2:**
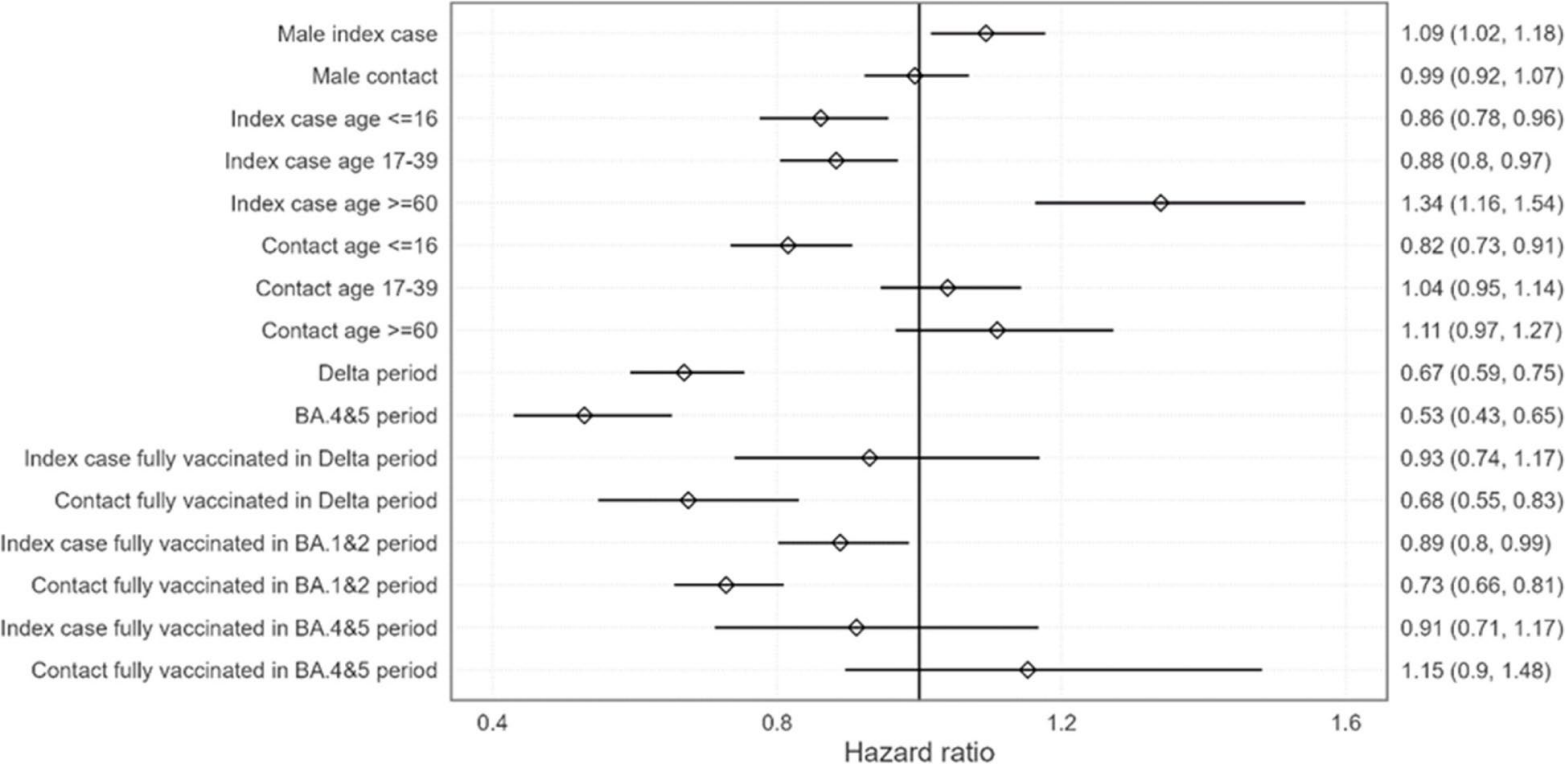
Modelled hazard ratios with fully vaccinated vaccination histories aggregated. Baseline is unvaccinated female case and contact of age 40–59 and in the omicron BA.1&2 period. Error bars and numbers in brackets represent 95% confidence intervals

Considering fully vaccinated individuals regardless of their vaccination schemes (one unique dose and a previous SARS-CoV-2 infection; or two doses regimen; or at least one booster dose), vaccination conferred protection to both index cases and contacts during the delta and omicron BA.1&2 periods in households (Fig. 2). This protection was significant except amongst cases during the delta period, likely driven by small sample sizes causing a large uncertainty in the estimate. Vaccine effectiveness against both transmission and infection during the BA.4&5 period was not statistically significant.

Gender had little impact on infection risk in households, with a slight increase in transmission risk for male cases. Considering age, transmission and infection risks were the lowest for the youngest cases and contacts (i.e. age ≤ 16).

**Fig. 3:**
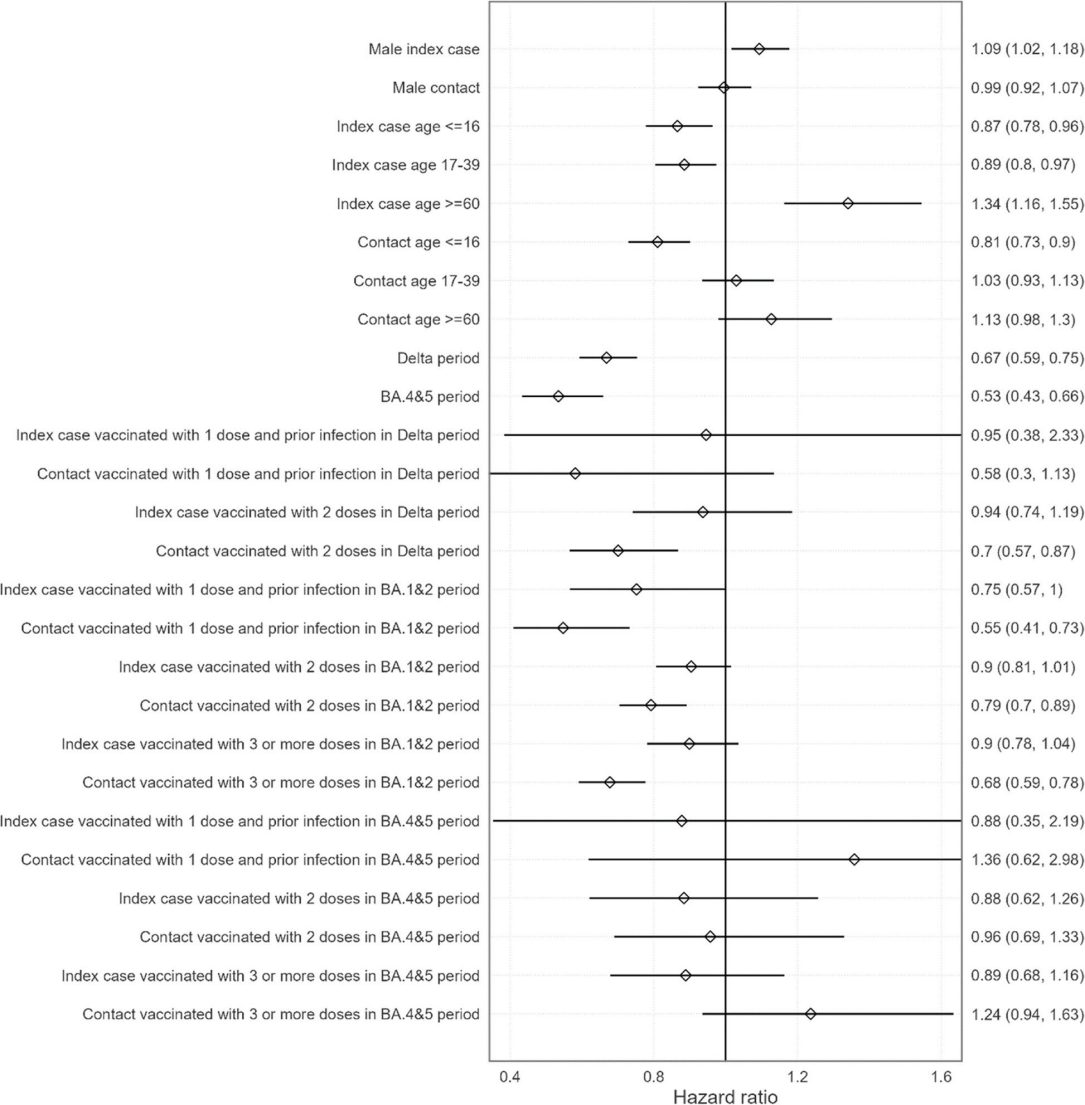
Modelled hazard ratios with fully vaccinated vaccination histories separated. Baseline is unvaccinated female case and contact of age 40–59 and in the omicron BA.1&2 period. Error bars and numbers in brackets represent 95% confidence intervals

Including disaggregate vaccination histories in the household model, the uncertainty increased due to the smaller group sample sizes involved (Fig. 3). However, the general picture remained the same. There was no significant effect of vaccine during the BA.4&5 period for both index cases and contacts. In the BA.1&2 period, contact vaccination was found to be significantly infection blocking regardless of fully vaccinated strategy. One vaccine dose with documented previous infection appeared to be the strongest, followed by three or more doses, followed by two doses. Case vaccination was significantly blocking transmission with one vaccine dose and a documented previous infection, but not for two nor three doses. Vaccinating contacts with two doses was found to be significantly infection blocking. All other effects were found to be not significant, taking into consideration the small sample sizes during the delta period. Gender, age, and variant effects were consistent with the aggregated model.

Adjusted vaccine effectiveness (aVE) in fully vaccinated (aggregate level) in households was low and declined as new variants emerged (Table 2). In contacts, aVE was calculated at 32% (17, 45) in delta; 27% (19, 34) in omicron BA.1&2; and − 15% (− 48, 10) in omicron BA.4&5. Considering index case vaccination status, aVE for transmission reduction was lower: 7% (− 17, 26) in delta; 11% (1, 20) in omicron BA.1&2; and 9% (− 17, 29) in omicron BA.4&5.

**Table 2:** Model outputs with vaccination histories aggregated in Monaco by VOC period between January 2021 and September 2022

Dominant variant	Vaccination status of case and contact	Variable value	Number of contact events	Number of infections	Hazard ratio — modelled	Vaccine effectiveness — modelled
Delta	Contact vaccination status	Fully vaccinated	365	137	0.68 (0.55, 0.83)	0.32 (0.17, 0.45)
Delta	Contact vaccination status	Unvaccinated	974	463	-	-
Delta	Index case vaccination status	Fully vaccinated	249	105	0.93 (0.74, 1.17)	0.07 (− 0.17, 0.26)
Delta	Index case vaccination status	Unvaccinated	1090	495	-	-
BA.1&2	Contact vaccination status	Fully vaccinated	2334	1217	0.73 (0.66, 0.81)	0.27 (0.19, 0.34)
BA.1&2	Contact vaccination status	Unvaccinated	1888	1121	-	-
BA.1&2	Index case vaccination status	Fully vaccinated	2114	1127	0.89 (0.8, 0.99)	0.11 (0.01, 0.2)
BA.1&2	Index case vaccination status	Unvaccinated	2108	1211	-	-
BA.4&5	Contact vaccination status	Fully vaccinated	501	246	1.15 (0.90, 1.48)	− 0.15 (− 0.48, 0.10)
BA.4&5	Contact vaccination status	Unvaccinated	311	121	-	-
BA.4&5	Index case vaccination status	Fully vaccinated	503	236	0.91 (0.71, 1.17)	0.09 (− 0.17, 0.29)
BA.4&5	Index case vaccination status	Unvaccinated	309	131	-	-

Numbers in brackets represent 95% confidence intervals

Considering individual vaccination histories, aVE was also decreasing alongside the SARS-CoV-2 variant emergence in households (Table 3). The greatest aVE was in contacts with a previous SARS-CoV-2 infection and a single vaccine dose during the omicron BA.1&2 period (45% [27, 59]), while the lowest were found in contacts with either three vaccine doses (aVE − 24% [− 63, 6]) or one single dose and a previous SARS-CoV-2 infection (aVE − 36% [− 198, 38]) during the omicron BA.4&5 period.

**Table 3:** Model outputs with vaccination histories separated in Monaco by VOC period between January 2021 and September 2022

Dominant variant	Variable	Variable value	Number of contact events	Number of infections	Hazard ratio — modelled	Vaccine effectiveness — modelled
Delta	Contact vaccination status	2	329	128	0.7 (0.57, 0.87)	0.3 (0.13, 0.43)
Delta	Contact vaccination status	0	974	463	-	-
Delta	Contact vaccination status	1p	28	9	0.58 (0.3, 1.13)	0.42 (− 0.13, 0.7)
Delta	Index case vaccination status	2	237	100	0.94 (0.74, 1.19)	0.06 (− 0.19, 0.26)
Delta	Index case vaccination status	0	1090	495	-	-
Delta	Index case vaccination status	1p	12	5	0.95 (0.38, 2.33)	0.05 (− 1.33, 0.62)
BA.1&2	Contact vaccination status	2	1091	588	0.79 (0.7, 0.89)	0.21 (0.11, 0.3)
BA.1&2	Contact vaccination status	0	1888	1121	-	-
BA.1&2	Contact vaccination status	3	1120	579	0.68 (0.59, 0.78)	0.32 (0.22, 0.41)
BA.1&2	Contact vaccination status	1p	123	50	0.55 (0.41, 0.73)	0.45 (0.27, 0.59)
BA.1&2	Index case vaccination status	2	1118	583	0.9 (0.81, 1.01)	0.1 (− 0.01, 0.19)
BA.1&2	Index case vaccination status	0	2108	1211	-	-
BA.1&2	Index case vaccination status	3	883	491	0.9 (0.78, 1.04)	0.1 (− 0.04, 0.22)
BA.1&2	Index case vaccination status	1p	113	53	0.75 (0.57, 1)	0.25 (0, 0.43)
BA.4&5	Contact vaccination status	3	336	179	1.24 (0.94, 1.63)	− 0.24 (− 0.63, 0.06)
BA.4&5	Contact vaccination status	0	311	121	-	-
BA.4&5	Contact vaccination status	2	151	60	0.96 (0.69, 1.33)	0.04 (− 0.33, 0.31)
BA.4&5	Contact vaccination status	1p	14	7	1.36 (0.62, 2.98)	− 0.36 (− 1.98, 0.38)
BA.4&5	Index case vaccination status	3	380	185	0.89 (0.68, 1.16)	0.11 (− 0.16, 0.32)
BA.4&5	Index case vaccination status	0	309	131	-	-
BA.4&5	Index case vaccination status	2	111	46	0.88 (0.62, 1.26)	0.12 (− 0.26, 0.38)
BA.4&5	Index case vaccination status	1p	12	5	0.88 (0.35, 2.19)	0.12 (− 1.19, 0.65)

*1p* a previous documented SARS-CoV-2 infection and one single vaccine dose

Numbers in brackets represent 95% confidence intervals

In a scenario comparing both fully vaccinated index cases and contacts with both unvaccinated index cases and contacts, aHR would be 0.63 (0.49, 0.81), 0.65 (0.58, 0.72) and 1.05 (0.81, 1.37) for delta, omicron BA.1&2 and omicron BA.4&5, respectively. Derived from this, the aVE would be 37% (19, 51), 35% (28, 42) and − 5% (− 37, 19) for delta, omicron BA.1&2 and omicron BA.4&5, respectively.

## Discussion

Based on a national programme prospectively investigating all contacts of a confirmed SARS-CoV-2 infection, we were able to quantify viral infection and direct and indirect vaccine effectiveness in real-life settings over a 14-month period. With a SAR of around 50%, household settings had the highest infection attack rates irrespective of index cases’ and contacts’ vaccination statuses, while occupational and school settings showed lower rates of infection. Such a difference may be due to infection pressure (duration and type of contact), as well as non-pharmacological measures such as mask wearing or social distancing, which are difficult to implement in households [29, 30].

Infection in household settings was higher than previously described: one meta-analysis estimated SAR out of 135 studies and reported 29.7% and 42.7% during the delta and omicron BA.1&2 periods, respectively [31]. Such differences may be explained by several factors: first, studies testing and collecting all contacts’ test results regardless of symptoms measured similarly high SAR, even in the pre-delta period [15]; second, Monaco is the most densely populated country in the world, with commuting workers doubling the local population every day, thus intensifying viral circulation. If the inclusion of asymptomatic contacts improves the generalisation of our findings, it also illustrates the intensity of viral circulation regardless of clinical presentation — and could lead to multiple importations into these settings.

In addition, the modelled aVE against infection was significant in delta and omicron BA.1&2, but low, 32% and 27%, respectively. Although this adjusted vaccine effectiveness is low, at the population level this indicates that a large number of vaccinated contacts would still be protected against infection during the delta and omicron BA.1&2 periods. The aVE for reducing transmission from index cases to contacts was substantially lower and was only significant during the omicron BA.1&2 period, though the lack of significance during the delta period may be caused by the smaller sample size. The aVE against both infection and transmission were statistically insignificant during the omicron BA.4&5 period, for all vaccination histories. The absence of vaccine impact during this latter period may be attributed to a combination of increased immune evasion for these variants and the fact that a large proportion of the population -including the unvaccinated—had by then been infected with SARS-COV-2 antigen by previous delta and in particular omicron BA.1&2 waves. Regardless of the vaccination scheme (i.e. one dose with a previous SARS-CoV-2 infection; two-dose regimen; or at least one booster dose), aVE was constantly calculated below 50% and reached its maximum in case of a previous SARS-CoV-2 infection, highlighting the importance of mucosal immunity in virus transmission [32–34]. Fully vaccinated contacts appeared to be more protected than cases (Fig. 2), and when analysing distinct vaccination schemes, the most protective strategy was provided amongst contacts with a single vaccine dose and a previous infection during the delta period (Fig. 3). During the omicron BA.1&2 period, fully vaccinated cases and contacts were significantly protected, and this protection was also conferred to the combination of a single dose and a previous infection. A booster dose was also providing a significant protection during the omicron BA.1&2 period, but only to contacts and not cases. There are only a few other studies that have screened all contacts in large prospective cohorts, and these similarly observed the limited impact of the vaccine on viral circulation [9–15]. However, many of these studies either did not adjust for important factors such as age, previous SARS-CoV-2 infections, number of vaccine doses including boosters, or inconsistently included both index cases and contacts without symptoms. Furthermore, none has studied the omicron BA.4&5 period. In the studies which adjusted for these factors, the authors similarly found significant aVEs for reducing both transmission and infection risk, but these findings were limited to the delta variant period [13, 14]. When adjusted for vaccination status, the delta and omicron BA.4&5 periods were found to have a lower risk of transmission and infection than the omicron BA.1&2 period. This may be due to the increased transmissibility of omicron BA.1&2 relative to delta, and the high levels of immunity generated during the omicron BA.1&2 period increasing population-level immunity during the omicron BA.4&5 period.

The strength of this analysis is the access to routine surveillance and immunization individual data on index cases and contacts for SARS-CoV2. This dataset includes several levels of disaggregation (age, gender, presence of symptoms, various dates) to produce vaccine effectiveness outputs in various settings. Initially, data quality check was operated manually; however, we defined processes and scripts to operate automated data quality checks and validation rules to improve the timeliness of analysis and reports. This process is reproducible and could be implemented on a continuous basis for real-time monitoring and evaluation of surveillance and immunization indicators for SARS-CoV.

By the end date of the study, the proportion of the population of Monaco that remained unvaccinated was 21%. In contrast, the proportion of unvaccinated index cases in this study is approximately 44%. This reflects the increased risk of becoming an index case if unvaccinated, which is a combination of increased risk of infection and increased risk of symptoms if infected (which increases the probability of the infection being detected and thus becoming an index case). Among contacts, the vaccination uptake differs by setting. In occupational settings, 26% of contacts were unvaccinated. This matches the population distribution, since the vaccine status of occupational contacts are independent of case vaccine status, and identification of contacts is independent of contact vaccine status. In household settings, however, the proportion of unvaccinated contacts was approximately 47%. This did not reflect the vaccine distribution among the general population, instead matching the vaccine distribution among index cases (and the case detection rate is expected to be higher in the unvaccinated part of the general population). This is because the vaccine status of household contacts will be dependent on the vaccine status of the corresponding index cases, since individuals within a single household will follow similar behaviour around vaccination. In the multivariate analysis, we adjust for the vaccine status of both index case and contact, which controls for this correlation in vaccine status.

Nevertheless, our analysis presents several limitations. First, positively tested contacts may have been infected by another index case than the one assumed due to other exposures outside the household. Our modelled analysis focused on households, where the likelihood to link index cases with contacts is higher than in other settings. Also, contacts who tested positive on the same day as their index case were considered co-primary cases, especially those with the same date of symptom onset (if any). Yet, we cannot exclude that some household contacts may be the actual index case. Second, only a small number of the 30,535 index cases and contacts were sequenced, so SARS-CoV-2 variant periods only represent an indicator of predominance. Further, we could not analyse each omicron subvariant separately, because of an insufficient number of sequenced cases and frequent co-circulation (e.g. SARS-CoV-2 omicron BA.1 and BA.2 co-circulated between February 2022 and March 2022, see Additional Fig. S2). Third, only contacts with a screening result were included in the analysis. Asymptomatic contacts may be less likely to get tested, which could lead to fewer vaccinated contacts getting tested due to reduced disease severity. It is therefore possible that we underestimated the number of infected contacts if vaccinated. Fourth, there was a large volume of missing data in the household analysis. This reduced the sample size which can be used in the statistical multivariate modelling. Since most missing data relates to the target variable, contact test result, or the main explanatory variable, vaccination status, we did not perform imputation. If the missingness is not at random, there is a risk that the exclusion of these data may introduce bias into the study. However, the majority of the missing values corresponded to contacts where the test was “not done”. Therefore, these are likely missing at random, and should not introduce much bias into the study. Most individuals removed due to missing vaccination data were due to poor data quality around vaccination dates. The collection of these data is likely uncorrelated with the infection status of the individual, so these are most likely missing at random. Therefore, the risk of bias from the missing data in this study is minimal. Another limitation of this study is the potential for self-selection bias. The statistical analysis focused on household settings, where high/low-risk behaviour is likely to have lower variance relative to external contacts. However, there may be some remaining behavioural biases. For example, if vaccinated individuals have milder symptoms, they may feel less inclined to isolate from household members prior to testing positive, which may lead to a higher risk of infection, reducing the observed protective effect of vaccination. On the contrary, unvaccinated individuals may be less health conscious, and therefore choose not to isolate from household members, which would bias the results in the other direction. Self-selection may also be present in the distribution of vaccines across the population. If vaccine uptake is higher in at-risk groups, this could affect the study. However, although individuals at higher risk may be more likely to become infected given an infectious contact, they may also undertake stricter isolation/protection measures, which could reduce the risk of becoming infected. These factors bias the results in opposite directions. We reduced this bias by adjusting for age, which accounts for a lot of multimorbidity associated with COVID-19 risk. However, without full multimorbidity data, we could not fully adjust for this. Last, we did not include the mRNA bivalent COVID-19 vaccine targeting both the original Wuhan-1 and omicron BA.4&5 strains, since our analysis ended prior to its launching in Monaco. Whether this vaccine is more efficient at limiting viral transmission and infection needs to be investigated, knowing that recent evidence reported limited neutralisation activity on the latest omicron subvariants [35–37].

The implication of such significant yet low direct and in particular indirect VE should be viewed from a public health perspective: although it was not known how effective COVID-19 vaccines were in preventing transmission some national campaigns promoted COVID-19 vaccine as a protective measure for “protecting others”, which may have created potential distrust, undermining population adherence to future immunisation recommendations [38]. Emphasising the utility of mRNA COVID-19 vaccines on severe disease and mortality and the role of non-pharmacological measures on transmission could help the population to better understand, and therefore accept, public health interventions [39–41].

## Conclusions

Our findings provide real-life evidence that the BNT162b2 mRNA vaccine provides low protection against SARS-CoV-2 infection and limited protection against transmission in households since the emergence of the delta variant. This effect has decreased with more recent variants. Future vaccination campaigns should emphasise the important role of COVID-19 vaccines in reducing severe disease and death among vulnerable population groups, rather than as a means to protect individuals from either transmitting or acquiring infection.

### Supplementary Information

Additional file 1: Table S1. Characteristics of index-cases and contacts by setting between July 2021 to September 2022 in Monaco. Figure S1: COVID-19 vaccination campaigns in Europe *versus* WHO communication on transmission and infection in 2021. Figure S2: Distribution of SARS-CoV-2 variants of concern between July 2021 and September 2022 in Monaco.

## Data Availability

The datasets used and/or analysed during the current study are available from the corresponding author on reasonable request.

## Abbreviations

BNT162b2: Pfizer BioNTech COVID-19 vaccine
CI: Confidence interval
COVID-19: Coronavirus disease
MCPHP: Monaco COVID-19 Public Health Programme
mRNA vaccine: Messenger ribonucleic acid vaccine
RT-PCR: Reverse-transcriptase polymerase chain reaction
RAT: Rapid antigen test
SAR: Secondary attack rate
SARS-CoV-2: Severe acute respiratory syndrome coronavirus 2
VE: Vaccine effectiveness
VOC: Variant of concern
