# Supplementary material for: How effective is the BNT162b2 mRNA vaccine against SARS-CoV-2 transmission and infection? A national programme analysis in Monaco, July 2021 to September 2022
Source: BMC Med. 2024 Jun 5;22:227. doi: 10.1186/s12916-024-03444-6 (PMC11155114; doi:10.1186/s12916-024-03444-6)

**How effective is the BNT162b2 mRNA vaccine against SARS-CoV-2 transmission and infection? A national programme analysis in Monaco, July 2021 to September 2022.**

*Additional file*

**Table S1.** Characteristics of index-cases and contacts by setting between July 2021 to September 2022 in Monaco

|  | **Household Setting** | | | **Occupational Setting** | | | **School Setting** | | |
| --- | --- | --- | --- | --- | --- | --- | --- | --- | --- |
|  | **Index Cases** | **Contacts Positive** | **Contacts Negative** | **Index Cases** | **Contacts Positive** | **Contacts Negative** | **Index Cases** | **Contacts Positive** | **Contacts Negative** |
| **Total, n (%)** | **4697** | **3584 (52%)** | **3371 (48%)** | **1094** | **423 (13%)** | **2868 (87%)** | **127** | **120 (6%)** | **1917 (94%)** |
| Tested with RT-PCR, n (%) | 4299 (92%) | 3316 (93%) | 2667 (79%) | 1056 (96%) | 402 (95%) | 2819 (98%) | 123 (97%) | 1899 (99%) | 117 (98%) |
| Tested with RAT, n (%) | 398 (8%) | 268 (7%) | 704 (21%) | 38 (4%) | 21 (5%) | 49 (2%) | 4 (3%) | 18 (1%) | 3 (3%) |
| 1 July 2021 – 11 December 2021 | 1091 (23%) | 705 (43%) | 930 (57%) | 378 (35%) | 162 (32%) | 1673 (44%) | 100 (79%) | 80 (66%) | 1663 (86%) |
| 12 December 2021 – 11 June 2022 | 2868 (61%) | 2459 (55%) | 1979 (45%) | 395 (36%) | 85 (17%) | 1100 (29%) | 21 (16%) | 14 (11%) | 244 (13%) |
| 12 June 2022 – 30 September 2022 | 725 (15%) | 418 (47%) | 480 (53%) | 321 (29%) | 253 (51%) | 1053 (27%) | 6 (5%) | 28 (2%) | 35 (2%) |
| Female, n (%) | 2370 (51%) | 1862 (54%) | 1710 (52%) | 399 (45%) | 199 (45%) | 1346 (44%) | 56 (50%) | 55 (51%) | 877 (52%) |
| Male, n (%) | 2289 (49%) | 1589 (46%) | 1548 (48%) | 485 (55%) | 244 (55%) | 1738 (56%) | 56 (50%) | 52 (49%) | 813 (48%) |
| Age <16, n (%) | 441 (9%) | 977 (27%) | 1052 (31%) | 9 (82%) | 1 | 54 (1%) | 106 (83%) | 106 (87%) | 1713 (88%) |
| Age 17-39, n (%) | 824 (17%) | 889 (25%) | 748 (22%) | 507 (46%) | 259 (52%) | 1710 (45%) | 16 (13%) | 13 (11%) | 151 (8%) |
| Age 40-59, n (%) | 910 (19%) | 1222 (34%) | 1246 (37%) | 530 (48%) | 223 (45%) | 1924 (50%) | 4 (3%) | 2 (2%) | 74 (4%) |
| Age 60-79, n (%) | 2520 (53%) | 488 (14%) | 327 (10%) | 47 (4%) | 17 (3%) | 135 (4%) | 1 (1%) | 1 (1%) | 4 (0%) |
| Presence of symptoms, n (%) | 4093 (87%) | 2186 (61%) | 190 (6%) | 353 (63%) | 244 (54%) | 3 | 1507 (63%) | 52 (43%) | 1 |
| Absence of symptoms, n (%) | 604 (13%) | 1407 (39%) | 3204 (94%) | 205 (37% | 212 (46%) | 3277 (100%) | 879 (37%) | 70 (57%) | 1941 (100%) |
| Known vaccination status, n (%) | 4509 (96%) | 3476 (97%) | 3160 (94%) | 820 | 411 | 1920 | 113 | 116 | 1451 |
| - Unvaccinated | 2036 (45%) | 1714 (49%) | 1379 (44%) | 298 (36%) | 194 (47%) | 410 (21%) | 98 (87%) | 103 (89%) | 1271 (87%) |
| - Fully vaccinated | 2290 (51%) | 1650 (48%) | 1656 (52%) | 488 (60%) | 189 (46%) | 1394 (73%) | 14 (12%) | 12 (10%) | 264 (11%) |
| - Unique dose previously infected | 101 (2%) | 73 (2%) | 93 (3%) | 12 (1%) | 4 (1%) | 19 (4%) | 1 (1%) | 0 | 14 (1%) |
| - Partially vaccinated | 82 (2%) | 39 (1%) | 32 (1%) | 22 (3%) | 24 (6%) | 37 (2%) |  | 1 (1%) | 2 |

**Figure S1.** COVID-19 vaccination campaigns in Europe *versus* WHO communication on transmission and infection in 2021

**Figure S2.** Distribution of SARS-CoV-2 variants of concern between July 2021 and September 2022 in Monaco


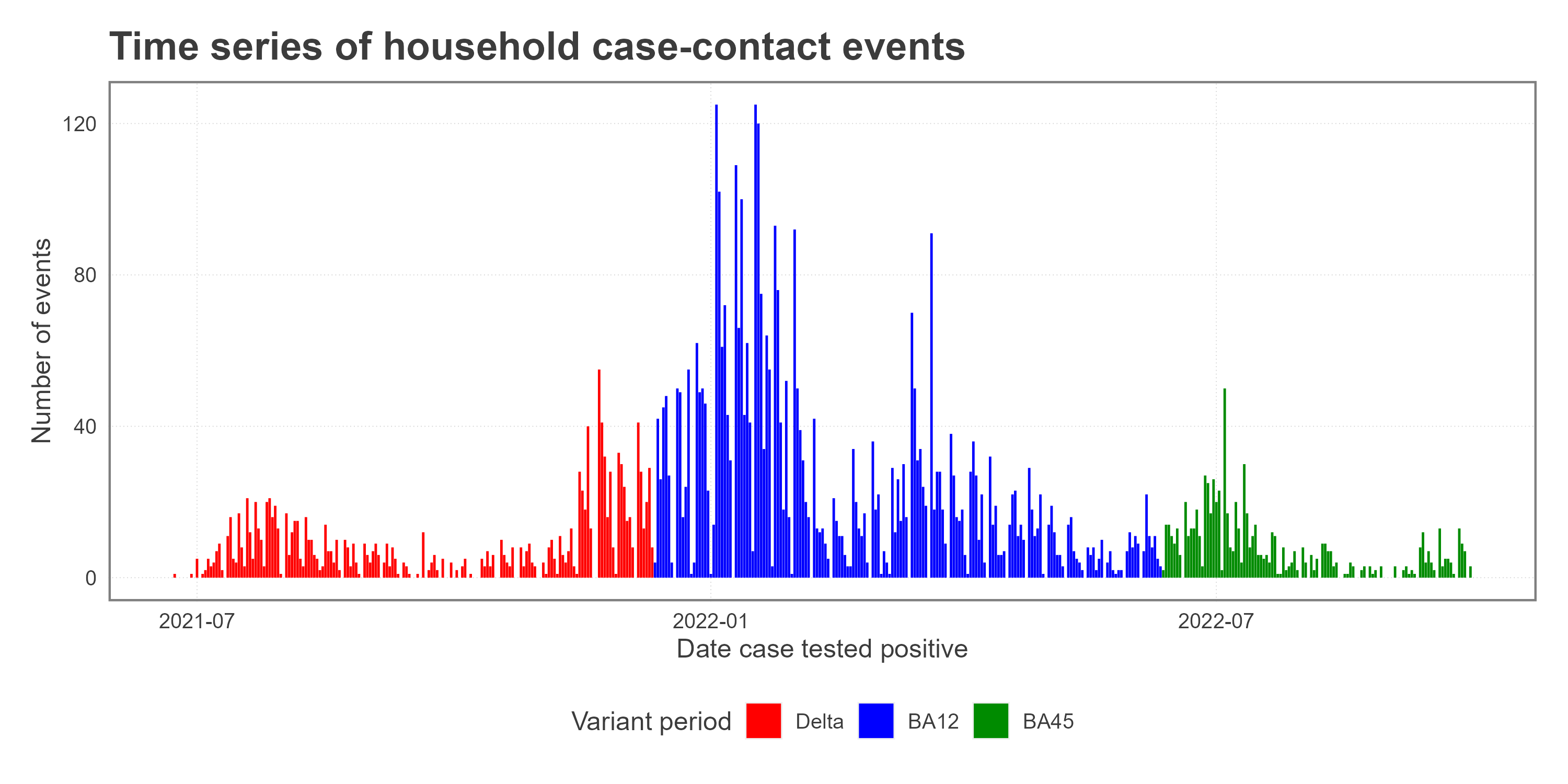


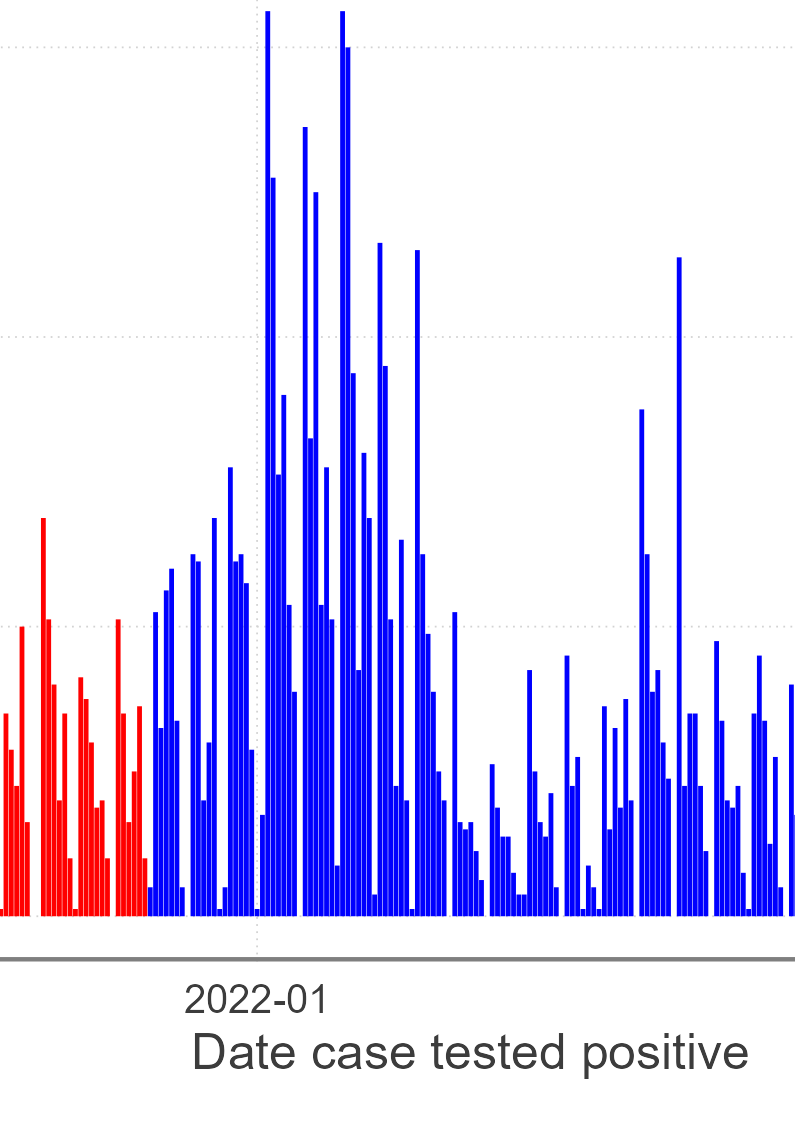

Supplement: Supplementary file 1 — Additional file 1: Table S1. Characteristics of index-cases and contacts by setting between July 2021 to September 2022 in Monaco. Figure S1: COVID-19 vaccination campaigns in Europe versus WHO communication on transmission and infection in 2021. Figure S2: Distribution of SARS-CoV-2 variants of concern between July 2021 and September 2022 in Monaco. [file 12916_2024_3444_MOESM1_ESM.docx]
